# Functional and Numerical Responses of *Tytthus chinensis* (Hemiptera: Miridae) to *Sogatella furcifera* (Hemiptera: Delphacidae)

**DOI:** 10.3390/insects16040339

**Published:** 2025-03-24

**Authors:** Qian Huang, Liping Long, Suosheng Huang, Biqiu Wu, Cheng Li, Yan Ling

**Affiliations:** Key Laboratory of Green Prevention and Control on Fruits and Vegetables in South China Ministry of Agriculture and Rural Affairs, Guangxi Key Laboratory of Biology for Crop Diseases and Insect Pests, Institute of Plant Protection, Guangxi Academy of Agricultural Science, Nanning 530007, China; huangqian2025@foxmail.com (Q.H.); longlp@sohu.com (L.L.); huangss@gxaas.net (S.H.); bqwu@gxaas.net (B.W.); lichengov@gxaas.net (C.L.)

**Keywords:** mirid bug, rice planthopper, predatory natural enemy, biological control, prey density, population dynamics, sustainable pest management

## Abstract

The predatory function and numerical responses of natural enemy insects are critical for evaluating their biological control potential, particularly in understanding pest suppression mechanisms and ecological adaptability. Here, we examined the predation capacity of *Tytthus chinensis* Stål (Hemiptera: Miridae) on *Sogatella furcifera* eggs under laboratory conditions (24 ± 1 °C, 12:12 h light:dark photoperiod, 75% humidity). Functional response experiments were conducted at different prey densities (3, 4, 10, 20, and 30 eggs/day), and life table parameters were established to evaluate the effects of prey density on the development, reproduction, and population dynamics of *T. chinensis*. The daily average predation of nymphs significantly increased with age, and their functional responses fitted the Holling Type II response. In the adult stage, the daily average predation of females was significantly higher than that of males, and males had a higher *a*/*T_h_* ratio, which reflected sex-specific predation strategies. Prey density significantly influenced survival, reproduction, and population dynamics. The minimum prey density required for population stability was 4 eggs/day, and survival and reproduction rates markedly increased at 10 eggs/day. At 30 eggs/day, females reproduced earlier, and the lifespan of males was shortened. These findings confirm the strong pest control ability of *T. chinensis* and highlight the critical role of *S. furcifera* egg density in regulating the population dynamics of *T. chinensis*.

## Simple Summary

Insects that prey on agricultural pests play a crucial role in protecting crops and reducing the reliance on chemical pesticides. Here, we focused on the mirid bug, Tytthus chinensis, an important natural enemy of the white-backed planthopper, a major pest of rice crops. In this study, we investigated its predation capacity at different life stages and how its population responds to varying prey densities under controlled laboratory conditions. Our results showed that predation rates increased as nymphs matured, with adult females consuming more pest eggs than males, though males fed more efficiently. When prey availability was low, population growth was slow, but higher prey densities significantly improved survival and reproduction, leading to rapid population expansion. These findings highlight the potential of the mirid bug as an effective biocontrol agent for managing rice planthopper populations. Utilizing such natural predators offers an ecofriendly and sustainable pest management strategy, reducing crop damage and minimizing pesticide use, which benefit both agriculture and the environment.

## 1. Introduction

The white-backed planthopper (*Sogatella furcifera* Horváth; Hemiptera: Delphacidae) is a major migratory pest that damages rice plants by sucking their sap, which leads to stunted growth or even plant death and causes significant yield losses [1]. Furthermore, *S. furcifera* is an important vector for viruses, such as the Southern rice black-streaked dwarf virus, which can potentially further exacerbate threats to rice production [2]. Currently, chemical control remains the most effective method for managing *S. furcifera* in the field. However, the prolonged use of chemical pesticides has resulted in the evolution of resistance to neonicotinoids and organophosphates in field populations of *S. furcifera* in some regions, which significantly undermines the efficacy of traditional chemical control methods [3,4,5,6].

The use of predatory insects for pest control can reduce the need for chemical pesticides, which minimizes pollution of the soil, water, and air; it also represents an environmentally friendly strategy for pest management [7,8]. In rice fields in China, the main natural enemies of *S. furcifera* that have been frequently reported include the egg parasitoid, *Anagrus nilaparvatae* Pang et Wang (Hymenoptera: Mymaridae) [9,10] and the predator mirid bug, *Cyrtorhinus lividipennis* Reuter (Hemiptera: Miridae) [11,12]. However, recent field surveys have shown that rice fields in Guangxi Province, China, serve as the first stopover site for annual migrations of rice planthoppers from Vietnam. In April, populations of migrating adult *S. furcifera* populations are established on rice seedlings, and the dominant natural enemy of *S. furcifera* is the mirid bug, *Tytthus chinensis* Stål (Hemiptera: Miridae). This insect is distributed in China, Thailand, and Iraq [13,14,15]. Its predatory behavior and life habits are similar to those of *C. lividipennis*. In rice fields, *T. chinensis* is a polyphagous predator that primarily preys on the eggs of rice planthoppers, including the brown planthopper (*Nilaparvata lugens*), white-backed planthopper, and small brown planthopper (*Laodelphax striatellus*). Previous studies have suggested that when *T. chinensis* and *C. lividipennis* coexist in rice fields, reciprocal intraguild predation may occur, which potentially reduces their effectiveness in controlling the brown planthopper [16]. *Tytthus chinensis* can more effectively control the brown planthopper under high-temperature conditions compared with *C. lividipennis*. At 38 °C, a female adult can consume an average of 40.49 brown planthopper eggs, suggesting that *T. chinensis* could be effective for the biological control of rice pests [17].

Functional and numerical responses are essential methods for scientifically and effectively evaluating the pest control potential of predatory natural enemies [18]. The functional response describes the predatory behavior of natural enemies at different prey densities, indicating how predation rates vary with prey density. It reflects the direct effect of predators on prey population density and can be used to assess their pest control capacity [18]. Functional responses are classified into three types: linear increase (Type I), a decelerating increase that reaches a plateau (Type II), and an S-shaped increase (Type III). Insect predators typically exhibit a Type II functional response, which is characterized by an initial increase in the predation rate with increasing prey density, followed by stabilization at higher prey densities [19]. *Tytthus chinensis* exhibits a Type II functional response when preying on brown planthoppers [13]. The numerical response describes how predator population dynamics (e.g., reproduction, mortality, and immigration/emigration) vary with prey density, reflecting an indirect response that explains how prey density influences predator population dynamics [20]. Numerical responses can be quantified through life table parameters (e.g., intrinsic rate of increase, net reproductive rate, generation time, and finite rate of increase), which comprehensively reflect the adaptability and growth potential of predator populations under different prey densities. These parameters collectively determine whether predators can rapidly establish populations in response to increasing prey densities, thereby effectively suppressing pests [21,22]. Therefore, life table parameters are valuable tools for assessing numerical responses and provide critical scientific support for developing biological control strategies and ecosystem management. By integrating studies on functional responses and life table parameters, a comprehensive evaluation of the pest control potential of predatory natural enemies can be achieved, offering theoretical support for sustainable pest management.

*Tytthus chinensis*, along with the white-backed planthopper (*Sogatella furcifera*), migrates to rice fields in April each year. However, no studies have reported the pest control potential of *T. chinensis* against *S. furcifera*. Here, we clarified the control effects of *T. chinensis* on *S. furcifera* by analyzing its functional and numerical responses; we also explored the relationship between *S. furcifera* density and the establishment of *T. chinensis* populations. The findings of this study have implications for the rearing and field release of *T. chinensis* in future biological control applications.

## 2. Materials and Methods

### 2.1. Insects and Plants

The rice variety Taichung Native 1 (TN1) was used to maintain populations of *S. furcifera* and *T. chinensis* in experiments. Rice seeds were germinated indoors and subsequently planted in plastic pots (19 cm in diameter × 14 cm in height). The pots with seedlings were then placed in insect cages to prevent contamination by other insect species. Rice plants that were 15–20 days old were used in feeding and oviposition experiments. *S. furcifera* and *T. chinensis* individuals were initially collected from rice fields at the Guangxi Academy of Agricultural Sciences, Guangxi Province, China, on 22 April 2023. Adult *S. furcifera* were reared in an oviposition cage containing potted TN1 rice plants. Rice plants infested with *S. furcifera* eggs from the colony were subsequently transferred into cages to serve as prey for *T. chinensis*.

### 2.2. Predation Daily Average Predation and Functional Response

Approximately 50 gravid female *S. furcifera* were introduced to tillering-stage rice plants for oviposition over a period of two days. The number of eggs laid was recorded under a stereomicroscope (Leica EZ4W, Wetzlar, Germany), and the tissue around the egg masses was gently teased open with a dissecting needle to ensure the eggs remained embedded within the stem. Once the desired number of eggs for the experiment was confirmed, the stem segments (ca. 1–3 cm) containing the eggs containing the eggs were cut and employed in the trials. Nymphs and adults of *T. chinensis* (one day after molting or emergence) were collected from the colony, starved for 5 h, and individually placed into small plastic cups (5 cm in diameter, 3.8 cm in height) with a layer of moist cotton at the bottom. A rice stem with egg masses at varying densities was placed on the cotton. Each cup was covered with a lid containing multiple small ventilation holes to ensure air exchange and maintain the experimental environment.

To determine the predation amount, 10, 30, and 60 eggs were provided for first-instar nymphs, second to fifth-instar nymphs, and adults, respectively. Twenty replications of each treatment were performed. After 24 h, *T. chinensis* was removed, and the number of eggs predated was recorded. Eggs that appeared shriveled were considered predated. The method for measuring the functional response was similar to that of the predation amount experiment, with additional prey densities provided in Table 1. Ten replications of each treatment were performed; after 24 h, the number of *S. furcifera* eggs predated was determined under a stereomicroscope. All experiments were conducted at 24 ± 1 °C, with a photoperiod of 12:12 (L:D) and a relative humidity of 75%.

### 2.3. Numerical Response

#### 2.3.1. Preparation of *Sogatella furcifera* Eggs

Tillering-stage rice plants were trimmed to remove yellow leaves and tillers; they were then rinsed thoroughly with clean water. Gravid *S. furcifera* females were introduced to the rice plants for oviposition. After 24 h, the rice stems containing eggs were collected, and the stems were dissected under a stereomicroscope. The portions of the rice stem with *S. furcifera* eggs were cut into small segments using scissors (ca. 1–3 cm). The number of eggs was observed under a stereomicroscope. The rice stem with *S. furcifera* eggs were placed in small plastic cups (5 cm in diameter, 3.8 cm in height) with moist cotton at the bottom based on the egg density specified in the experimental design. These were used for subsequent experiments.

#### 2.3.2. Preparation of *Tytthus chinensis* Nymphs

Adult *T. chinensis* males and females from the colony were introduced onto tillering-stage rice plants for oviposition, along with gravid *S. furcifera* females as prey. The oviposition date was recorded, and nymphs with a uniform egg developmental period of 7 days were used for the experiment. On the 7th day, the rice plants were shaken to dislodge first-instar nymphs of *T. chinensis* onto a white porcelain plate. The newly hatched nymphs were removed using a fine brush and placed into small plastic cups containing varying densities of *S. furcifera* eggs (3, 4, 10, 20, and 30 eggs per day). Each treatment included 30 nymphs, and the hatching time of nymphs was recorded. Eggs corresponding to the specified densities were replenished daily for *T. chinensis* nymphs, and their molting status was observed and recorded until adult emergence. After emergence, adult males and females were paired and placed into graduated cylinders (2.5 cm in diameter, 15 cm in height). Each cylinder contained a tillering-stage rice plant segment approximately 15 cm long, with roots wrapped in moist cotton to maintain humidity. The rice stem was used for oviposition, and varying densities of *S. furcifera* eggs (6, 8, 20, 40, and 60 eggs per day) were provided as prey for paired male and female adults. The rice stems used for oviposition were replaced every two days, and the number of eggs laid by each female was observed under a stereomicroscope. The lifetime of the adult was recorded upon death.

#### 2.3.3. Observation of the Next Generation

A total of 32 eggs laid by *T. chinensis* females were randomly selected from each treatment (only 32 eggs were available because of limited egg production in the 4-egg density treatment; see Results Section). The hatching rate was calculated as the proportion of eggs that successfully hatched, and the egg developmental period was recorded for each treatment.

#### 2.3.4. Statistical Analysis

The least significant difference (LSD) method in one-way ANOVA was used to analyze the mean daily predation, developmental duration of next-generation eggs, and egg hatching rate of *T. chinensis* across treatments. Before ANOVA, normality and homogeneity of variances were tested using the Shapiro-Wilk and Levene’s tests, respectively. Since egg hatching rate is a proportion and violated normality, an arcsine square root transformation was applied. All transformed data met ANOVA assumptions. Statistical significance was set at *p* = 0.05, and analyses were performed in IBM SPSS Statistics 19.0.

Life table data for *T. chinensis* reared under varying *S. furcifera* egg densities were analyzed based on age-stage, two-sex life table theory [23,24,25]. The analysis was conducted using the TWOSEX-MSChart program [26], a specialized tool for life table parameter estimation based on age-stage and two-sex data. The paired bootstrap test [27] was used to calculate key life history parameters of *T. chinensis*, including the developmental duration of the five nymphal instars (first to fifth instar), the pre-oviposition period (the time from female adult emergence to first egg-laying), fecundity, male and female longevity, and population parameters such as the intrinsic rate of increase and finite rate of increase.

Different life table parameters of *T. chinensis* reared under varying *S. furcifera* egg densities were calculated, including:

Age-stage specific survival rate *S_xj_* (the survival rate of *T. chinensis* in age *x* and stage *j*, Equation (1));

(1) $$S_{xj}=\frac{n_{xj}}{n_{01}}$$

where *n_xj_* indicates the number of individuals surviving to age *x* and stage *j*, and *n*_01_ denotes the initial population size recorded at the beginning of the study as presented in the life table.

The age-specific survival rate, *l_x_*, is the probability that a newly hatched 1st-instar nymph survives to age *x* (Equation (2)).

(2) $$l_{x}=\sum_{j=1}^{m}s_{xj}$$

where *m* is the number of the life stage.

The age-stage-specific fecundity, *f_xj_*, is the mean fecundity of female individuals of age *x* and stage *j* (Equation (3)).

(3) $$f_{xj}=\frac{E_{xj}}{n_{xj}}$$

where *E_xj_* denotes the total number of eggs produced by the individuals *n_xj_*.

The age-specific fecundity, *m_x_*, is the mean fecundity of individuals at age *x* (Equation (4)).

(4) $$m_{x}=(\sum_{j=1}^{m}s_{xj}f_{xj})/\sum_{j=1}^{m}s_{xj}$$

The age-stage life expectancy, *e_xj_*, is the remaining lifetime expected for an individual of age *x* and stage *j* (Equation (5)).

(5) $$e_{xj}=\sum_{i=x}^{\infty }\sum_{r=j}^{k}s_{iy}^{\prime }$$

where $s_{iy}^{\prime }$ is defined as the probability of an individual at age *x* and stage *j* surviving to reach age i and stage y, under the assumption that $s_{iy}^{\prime }=1$.

The aim of this experiment was to examine the effect of prey density on the life table parameters of *T. chinensis*. Since the egg stage of *T. chinensis* does not participate in predation, the calculations of the aforementioned parameters were performed at the start of the first-instar nymph stage.

The net reproductive rate, *R*_0_ (the total number of offspring generated by an individual over its lifetime, Equation (6)); intrinsic rate of increase, *r* (the per capita rate of population growth under ideal environmental conditions with a stable age distribution, Equation (7)); finite rate of increase, *λ* (the geometric population growth rate per unit time, Equation (8)); and the mean generation time, *T* (the average time interval between the birth of a parent and the birth of its offspring, Equation (9)) were also calculated using the program TWOSEX-MSChart. As these population ecological parameters reflect processes involving the entire life cycle, their calculations were initiated from the egg stage.

(6) $$R_{0}=\sum_{x=0}^{\infty }l_{x}m_{x}$$

(7) $$\sum_{x=0}^{\infty }e^{-r(x+1)}l_{x}m_{x}=1$$

(8) $$\lambda =e^{r}$$

(9) $$T=(\ln R_{0})/r$$

The functional response data were fitted using the Holling Type II model, based on our confirmation through logistic regression analysis that *T. chinensis* exhibits a Type II functional response when preying on *S. furcifera* eggs [19]. The handling time (*T_h_*) represents the total time available for the predator to search for prey during the experiment (1 day); *a* is the attack rate. The theoretical daily maximum prey consumption was calculated as 1/*T_h_*, which represents the maximum number of prey a predator can consume per day under ideal conditions. The ratio of *a*/*T_h_* is a comprehensive indicator of a predator’s predation capability, reflecting both the attack rate and handling efficiency. A Type II response was estimated using the following disc equation (Equation (10)):

(10) $$N_{a}=\frac{aTN_{0}}{1+aT_{h}N_{0}}$$

The relationship between the oviposition of *T. chinensis* and the egg density of *S. furcifera* was modeled using the following hyperbolic equation (Equation (11)) [28], as follows:

(11) $$y=\frac{ax}{b+x}$$

where *a* is the asymptote, representing the natural logarithm of the maximum total oviposition; *b* is the prey density required to reach half the asymptote; and *y* is the natural log-transformed total oviposition of *T. chinensis* at various densities of *S. furcifera* eggs (*x*).

The data for Equations (10) and (11) were analyzed using SPSS 19.0. Both models were fitted, and their parameters were estimated through nonlinear regression analysis.

## 3. Results

### 3.1. Daily Average Predation

**Figure 1:**
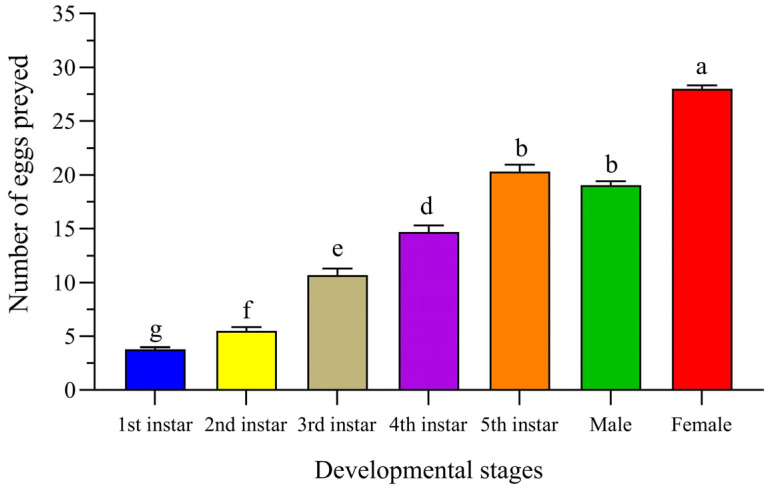
The daily number of eggs *Sogatella furcifera* preyed upon by *Tytthus chinensis.* Different letters above each bar indicate significant differences between stages using one-way ANOVA, LSD test (*F*_6,133_ = 328.97, *p* < 0.05).

The daily average predation of *T. chinensis* on *S. furcifera* eggs under a sufficient egg supply is presented in Figure 1. The figure shows that the daily average predation increased with the developmental stage. Significant differences were observed among the 1st–5th instar nymphs, adult females, and adult males (F_6,133_ = 328.91, *p* < 0.01). The daily average predation of the first-, second-, third-, fourth-, and fifth-instar nymphs was 3.80 ± 0.18, 5.50 ± 0.35, 10.70 ± 0.60, 14.70 ± 0.61, and 20.30 ± 0.67 eggs, respectively. During the adult stage, females exhibited significantly higher daily predation than males, with averages of 28.00 ± 0.35 and 19.05 ± 0.36 eggs, respectively.

### 3.2. Functional Response

The functional response parameters of *T. chinensis* preying on *S. furcifera* eggs are shown in Table 2. The functional responses of all developmental stages fitted well with the Holling Type II model. During the nymphal stage, the handling time decreased as the instar stage increased. The fifth-instar nymph had the highest attack rate (*a*) and the shortest handling time (*T_h_*), which were 3.108 and 0.029 days, respectively. During the adult stage, male adults had a higher attack rate (11.354) compared with female adults.

The ratio of *a*/*T_h_* is a comprehensive indicator of a predator’s predation capability. Male *T. chinensis* adults had the strongest predation capability on *S. furcifera* eggs, with an *a*/*T_h_* ratio of 214.23, followed by the fifth-instar nymphs, with a ratio of 107.17.

### 3.3. Effects of Different Prey Densities on the Survival Rate and Age-Stage-Specific Survival Rate of T. chinensis

**Figure 2:**
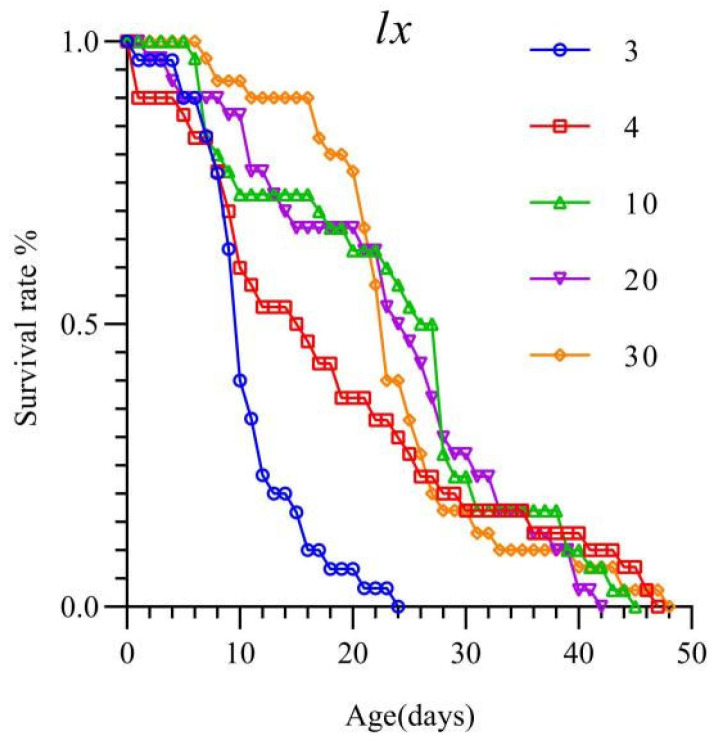
Survival rates of *Tytthus chinensis* under different prey densities of *Sogatella furcifera* eggs.

The survival rate of *T. chinensis* preying on different densities of *S. furcifera* eggs is shown in Figure 2. The figure indicates that the survival rate decreased to 40% by day 10 at a prey density of 3 eggs/day, and all individuals died by day 24. At prey densities of 4, 10, 20, and 30 eggs/day, the survival rate was 50% on days 15, 26, 24, and 23, respectively, with all individuals dying on days 47, 45, 42, and 48, respectively. These results suggest that a prey density of 3 eggs/day is unsuitable for the survival of *T. chinensis*.

**Figure 3:**
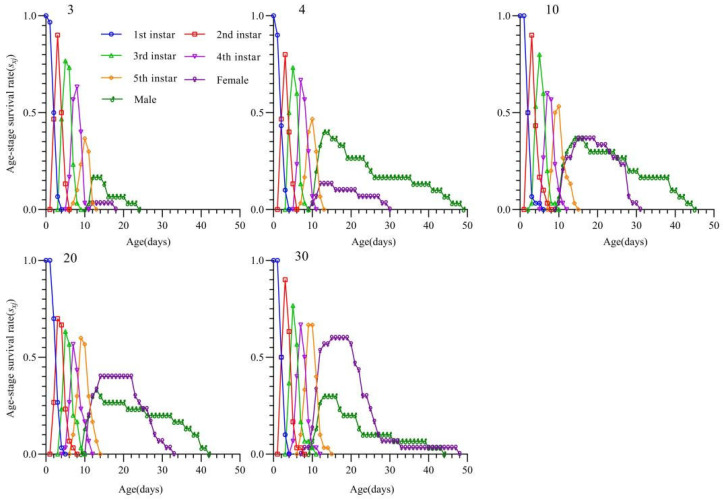
Age-stage survival rates (*S_xj_*) of *Tytthus chinensis* under different prey densities of *Sogatella furcifera* eggs.

The age-stage survival curves are shown in Figure 3. At prey densities of 3 and 4 eggs/day, the survival rate of *T. chinensis* decreased below 50%, starting from the fifth-instar stage. At a density of 3 eggs/day, both male and female adults had lower survival probabilities compared with that in other treatments, and the survival probabilities of female adults were even lower than those of males. Starting from a prey density of 10 eggs/day, the survival probability of female adults exceeded that of males and increased as prey density increase.

### 3.4. Reproductive Parameters of T. chinensis Under Different Prey Densities

**Figure 4:**
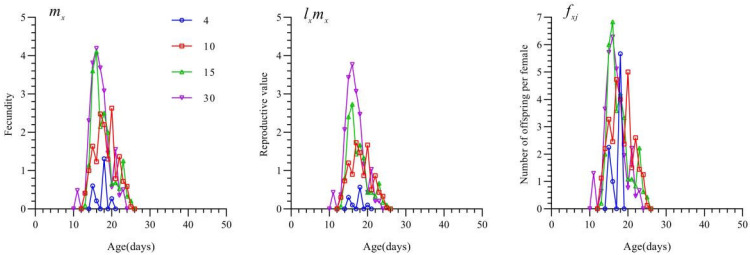
Fecundity(*m_x_*), reproductive value(*l_x_m_x_*), and age-specific fecundity(*f_xj_*) of *Tytthus chinensis* under different densities of *Sogatella furcifera* eggs.

The fecundity, reproductive value, and age-specific fecundity curves of *T. chinensis* under different densities of *S. furcifera* eggs are shown in Figure 4. At a prey density of 3 eggs/day, no eggs were laid by female adults; thus, reproductive-related curves were not available for this density.

The *m_x_* fecundity curves indicated that at prey densities of 4, 10, 20, and 30 eggs/day, female adults began reproducing on days 15, 13, 13, and 11, respectively, and reached peak fecundity on days 18, 20, 16, and 16, respectively. The duration of oviposition was shorter at a density of 4 eggs/day than at other densities, and intermittent reproductive pauses were observed. The maximum *m_x_* value (4.19) was observed at 30 eggs/day.

The *l_x_m_x_* curve, which represents the product of the survival rate and fecundity, showed that the peak value increased at higher prey densities. The *f_xj_* curve followed a pattern similar to the *m_x_* curve, but the total fecundity was higher at a prey density of 20 eggs/day (6.833) than at a prey density of 30 eggs/day.

**Figure 5:**
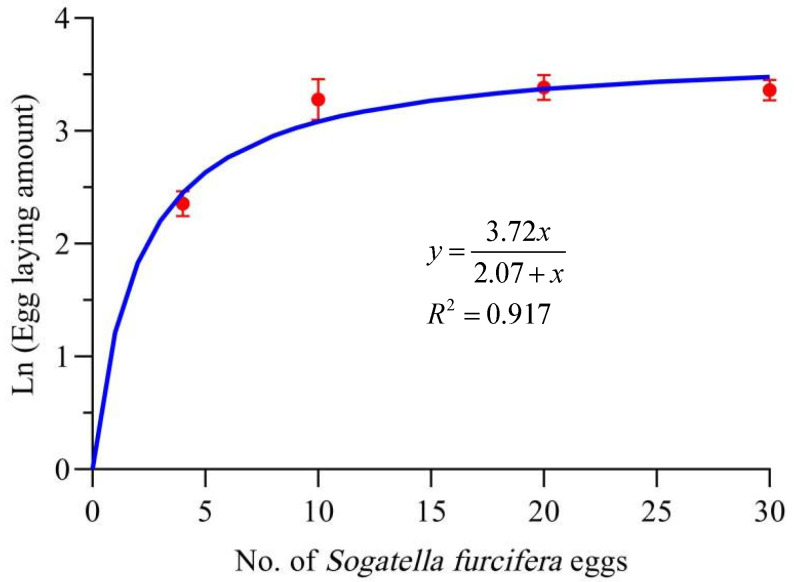
Relationship between the natural logarithm of *Tytthus chinensis* egg production and the density of *Sogatella furcifera* eggs. The dots represent observed data, and the error bars indicate the standard error of the mean.

The natural logarithm of the oviposition of female adult *T. chinensis* (y) was strongly correlated with the density of *S. furcifera* eggs (*x*) (*R*^2^ = 0.917, Figure 5). The fecundity of *T. chinensis* increased at higher prey consumption rates and tended to stabilize when prey consumption exceeded 10 *S. furcifera* eggs/day. The asymptote(*a*) was 3.72, and the prey density at half the asymptote (*b*) was 2.07. Therefore, the maximum fecundity (*e*^3.72^) of *T. chinensis* was 41.26 eggs, and the prey density required to elicit half the maximum fecundity (*e*^2.07^) was 7.92 *S. furcifera* eggs.

### 3.5. Effects of S. furcifera Egg Density on the Growth and Development of T. chinensis

As shown in Table 3, the increase in *S. furcifera* egg density had no significant effect on the pre-adult duration, pre-oviposition period of female adults, or adult lifespan of *T. chinensis*. However, a decrease in egg density significantly affected the oviposition of female adults and the hatching rate of the next-generation eggs. Females oviposited 8.00 ± 2.80 eggs at a prey density of 4 eggs/day, which was significantly lower than that at other densities, and the hatching rate of the next generation was also significantly lower at a prey density of 4 eggs/day than at higher prey densities.

### 3.6. Effects of S. furcifera Egg Density on the Population Dynamics of T. chinensis

As shown in Table 4, the net reproductive rate (*R*_0_), mean generation time (*T*), intrinsic rate of increase (*r_m_*), and finite rate of increase (λ) of *T. chinensis* varied significantly under different *S. furcifera* egg densities. *R*_0_, *r_m_*, and *λ* were significantly lower at a prey density of 4 eggs/day than at other densities. At a density of 10 eggs/day, *r_m_* and λ were significantly lower, while the *T* was significantly longer at a density of 10 eggs/day compared with a density of 30 eggs/day. No significant differences were observed in these parameters between prey densities of 20 and 30 eggs/day.

The female-to-male ratio increased with the prey densities. At a density of 4 eggs/day, the female ratio was 25%; at 10 eggs/day, it was 50%; and at densities of 20 and 30 eggs/day, it reached 66.67%.

**Figure 6:**
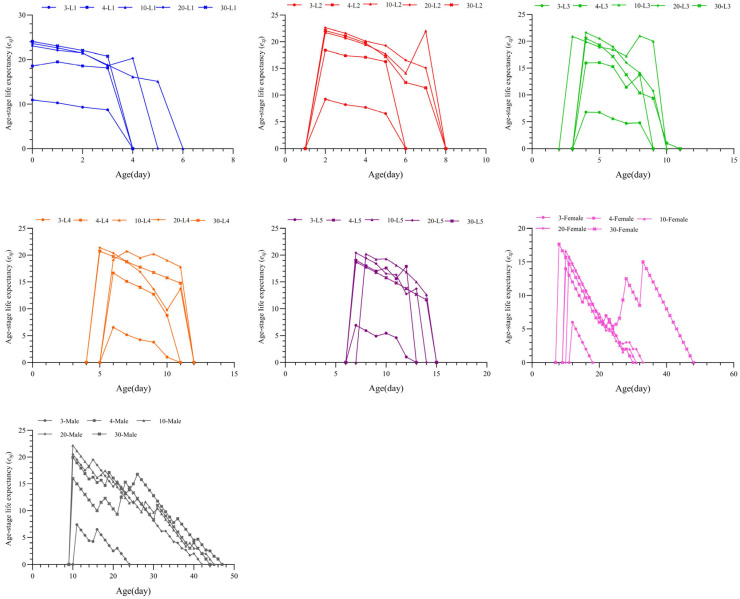
Age-stage life expectancy (*e_xj_*) of *Tytthus chinensis* under different densities of *Sogatella furcifera* eggs.

The age-stage life expectancy (*e_xj_*) reflects the survival and developmental potential of *T. chinensis* at different stages. When the *S. furcifera* egg density was 3 eggs/day, the initial age-stage life expectancy (*e_xj_*) values for all stages of *T. chinensis* were lower than those at other densities (Figure 6). The initial *e_xj_* value for adult males was greater at a density of 4 eggs/day than at 30 eggs/day, whereas the values at 4 eggs/day were lower than those at any other densities at other stages. When the egg density increased to 10, 20, or 30 eggs/day, the initial *e_xj_* values for most stages overlapped, and no distinct differences were observed; however, the values for adult males remained lower at 30 eggs/day remained lower than at 10 and 20 eggs/day. At a density of 30 eggs/day, the *e_xj_* values of females were similar to those of males; at lower densities, males generally exhibited higher *e_xj_* values than females.

## 4. Discussion

Studying the predation function of natural enemy insects is crucial for assessing their potential efficacy for biological control, as well as for understanding their pest control mechanisms and ecological adaptability [29,30]. We investigated the predatory capacity and functional responses of *T. chinensis* to *S. furcifera* eggs and focused on characterizing its predatory performance and ecological roles across different developmental stages. The results reveal differences in the predatory behavior of *T. chinensis* between different nymphal instars and between female and male adults. When *S. furcifera* eggs were abundant, the amount of prey consumed by *T. chinensis* nymphs increased with age, and the daily average predation increased from 3.80 ± 0.19 eggs for first-instar nymphs to 20.30 ±0.67 eggs for fifth-instar nymphs. The functional responses of all developmental stages were well-fitted to the Holling Type II model. In the adult stage, females exhibited a daily average predation of 28.00 ± 0.35 eggs when prey availability was high, which was significantly higher than the 19.05 ± 0.36 eggs consumed by males. This can be attributed to the higher nutritional demands of females to support their reproductive activities. As noted by Hosseini et al. (2019), female variegated lady beetles, *Hippodamia variegata* Goeze, typically consume more prey (cotton aphid, *Aphis gossypii*) to support egg development in their ovaries, which reflects their greater investment of time and energy in predation to meet their physiological needs [31]. Moreover, Lv et al. emphasized that female predatory mites (*Neoseiulus barkeri* Hughes) may consume prey with high nutritional quality through selective predation, which supports their reproductive activities [32]. However, although male adults had a lower daily consumption, they had a higher *a*/*T_h_* ratio than females in the functional response. This result is consistent with the findings of Pimsamarn et al. [13], showing a similar trend in the predation of *T. chinensis* on *N. lugens* eggs. This may reflect an adaptive strategy of males, which provides them with a competitive advantage [33]. As shown by Rashedi et al. [34], male predatory bugs (*Orius albidipennis*) have a stronger predation capacity on *Aphis fabae* compared with females, and the most pronounced differences were observed in the attack rate. More frequent attacks enhanced their competitive ability, which may enhance their predation performance and help attract females during the reproductive period [35]. The predation results indicated that all nymphal stages of *T. chinensis* could prey on *S. furcifera* eggs, and older nymphs and both male and female adults had stronger predation abilities; *T. chinensis*, thus, had a significant control effect on *S. furcifera*.

The colonization process of predatory insects in specific ecological areas is influenced by multiple factors, including environmental variables such as soil type and vegetation cover, as well as meteorological factors like temperature and humidity [36,37,38]. A particularly important factor is the abundance of prey, which has a significant impact on the colonization of predatory insects, given that it directly affects their survival, reproduction, and the stability of their ecological niche within the ecosystem [21,39]. Our findings indicate that the density of *S. furcifera* eggs had a significant effect on the survival and reproduction of *T. chinensis*. When the egg density is low, the survival and reproductive rates of *T. chinensis* are constrained. Recent research shows that at an egg density of only 3 eggs/day, the survival rate of *T. chinensis* significantly decreases to 40% by the 10th day, and all individuals die within 24 days. Furthermore, female adults do not lay eggs, and the life expectancy (*e_xj_*) of individuals across all nymphal stages is significantly lower at a density of 3 eggs/day than at other densities. This result suggests that a low egg density is insufficient to meet the survival needs of *T. chinensis*, which affects its reproductive capacity. This finding is consistent with those of Agarwala et al. showing that the density of the ladybird beetle *Menochilus sexmaculatus* depends on aphid populations, which emphasizes the reliance of predator populations on prey abundance and the limiting effect of prey availability on the survival and reproductive capacity of predators [40]. At an egg density of 4 eggs/day, the survival rate of *T. chinensis* significantly increased, and females were able to lay eggs; however, egg production was significantly lower at an egg density of 4 eggs/day than at higher densities (10, 20, and 30 eggs/day), and reproduction temporarily paused. This suggests that under resource scarcity, female *T. chinensis* may forego reproduction to enhance survival. This is consistent with the findings of Jervis et al., which showed that natural enemy insects adjust their reproductive strategies to ensure survival at low prey densities. [41]. Tangkawanit et al. reported that *C. lividipennis*, which has similar biological traits as *T. chinensis*, requires 5 planthopper eggs per day to develop into adults, and 10 eggs per day for reproduction [28]. By comparison, *T. chinensis* requires only three eggs per day to develop into adults, and reproduction can occur when four eggs per day are provided. Therefore, *T. chinensis* populations can be sustained under low prey densities.The fecundity and age-stage life expectancy of female adults are significantly higher at egg densities of 10 and 20 eggs/day than 4 eggs/day, and the survival rate (*S_xj_*) of females surpassed that of males. Additionally, egg production at 10 and 20 eggs/day did not significantly differ from that at 30 eggs/day. This result is consistent with the findings of Batool et al. (2014) showing that increases in the density of grain moth (*Sitotroga cerealella*) eggs significantly enhanced the survival rate and fecundity of the predator green lacewing, *Chrysoperla carnea* [42]. Further analysis revealed that when the egg density increased to 30 eggs/day, the fecundity of female adults peaked (*m_x_* = 4.19), but this was accompanied by an earlier onset of reproduction. This early reproduction strategy optimized early output but led to an uneven reproductive distribution, resulting in a lower peak value of *f_xj_* (6.28) compared to 20 eggs/day (6.83). This phenomenon may occur because predators accelerate their reproductive rhythm under high prey density to quickly exploit abundant resources. Similar early reproduction strategies under resource-abundant conditions have been reported in other predatory insects. Hemptinne et al. observed that predatory ladybirds adjust their breeding strategies in response to aphid density to maximize reproductive success [43]. The age-stage life expectancy (*e_xj_*) and overall lifespan of male adults were lower at an egg density of 30 eggs/day than at 4 eggs/day. In the experiment, *T. chinensis* adults were observed to mate multiple times. Based on this observation, the reduced lifespan of males may be attributed to the increased mating frequency required by females to maximize reproductive success under high prey densities. Frequent mating likely results in excessive energy expenditure in males, which reduces their survival rate. These findings are similar to those of Wang et al., who showed that increased mating frequency significantly shortened the lifespan of male diamondback moths, *Plutella xylostella* [44].

Our findings revealed the effects of *S. furcifera* egg density on the population dynamics of the natural enemy *T. chinensis*. The net reproductive rate (*R*_0_), intrinsic rate of increase (*r_m_*), and finite rate of increase (*λ*) of *T. chinensis* were significantly lower at an egg density of 4 eggs/day than at higher egg densities. This suggests that low prey density restricts the population growth potential of the predator; however, an *R*_0_ value greater than 1 indicates that *T. chinensis* can still maintain its population at this density. At higher densities (20 and 30 eggs/day), the population parameters tended to stabilize, with no significant differences observed between the two densities, indicating that *T. chinensis* shows a saturating numerical response to *S. furcifera* eggs. This result is consistent with the findings of Madahi et al., who showed similar trends in the population parameters of the aphidophagous midge, *Aphidoletes aphidimyza*, fed varying densities of the cotton aphid, *Aphis gossypii* [45]. Further observations revealed that increases in egg density resulted in significant changes in the female-to-male ratio, rising from 25% at low density to 66.67% at high density. The age-stage survival rate curves (Figure 3) indicate that this shift is primarily attributed to improved survival rates of female adults. In addition, the egg density of *S. furcifera* significantly affected the hatching rate of the subsequent generation of *T. chinensis*. Previous studies have demonstrated that increased adult nutritional intake can significantly enhance the egg-hatching rate in the next generation [46,47,48]. Therefore, higher prey densities might provide sufficient nutrition, which increases the proportion of fertilized eggs in *T. chinensis*, ultimately improving egg-hatching rates.

Based on our findings, we recommend releasing *T. chinensis* just before the peak oviposition period of *S. furcifera* to ensure sufficient prey resources for rapid population establishment. At an egg density of 10 eggs/day, *T. chinensis* exhibits significantly higher survival and reproduction rates, leading to accelerated population growth. Releasing the predator at this stage effectively suppresses pest populations during their early development, maximizing biological control efficiency. In artificial rearing, we recommend maintaining an *S. furcifera* egg density of 10–20 eggs/day to balance *T. chinensis* population growth and resource efficiency, as this range supports rapid reproduction while mitigating trade-offs such as reduced male lifespan and uneven reproductive distribution at higher densities. Optimizing rearing density ensures a stable *T. chinensis* supply for biological control programs while minimizing prey production costs. By integrating optimal field release timing with rearing density adjustments, this study provides valuable guidance for the effective application of *T. chinensis* in rice ecosystems.

However, this study has certain limitations that require consideration. In the experimental design, the daily egg density provided during the adult stage was based on the combined feeding requirements of both male and female adults by taking their mating needs into account. Due to predatory competition between males and females and the superior predation ability of males, females may have experienced reduced food availability under low egg densities. This could have led to an inaccurate estimation of the actual prey requirements for single-sex adults. Furthermore, to more comprehensively assess the biocontrol potential of *T. chinensis*, future studies should also measure the life table parameters of *S. furcifera* under the same experimental conditions to more accurately compare the population growth dynamics of the predator and its prey, thereby providing a more comprehensive evaluation of *T. chinensis* as a biological control agent.

## 5. Conclusions

The results of this study demonstrated that *T. chinensis*, a polyphagous predator of rice planthopper eggs, can effectively control *S. furcifera*. The minimum egg density required to sustain the *T. chinensis* population is 4 eggs/day. At an egg density of 10 eggs/day, survival and reproductive rates increased significantly, leading to rapid population growth. Higher egg densities (e.g., 20 and 30 eggs/day) further enhanced population parameters, but 30 eggs/day may involve trade-offs such as reduced male lifespan and uneven reproductive distribution. Therefore, 10 eggs/day is sufficient to support rapid population growth of *T. chinensis*.

To maximize the effectiveness of biological control, the optimal timing for releasing *T. chinensis* in the field should be just prior to the peak oviposition period of *S. furcifera*. This strategy ensures that prey availability is sufficient to support the establishment and maintenance of a stable *T. chinensis* population. In artificial rearing environments, egg density can be adjusted based on production objectives to promote population stability and development.

## Figures and Tables

**Table 1:** Prey density settings for functional response experiments of *Tytthus chinensis* at different developmental stages on *Sogatella furcifera* eggs.

Treatment	Number of *Sogatella furcifera* Eggs (Eggs/Unit)						
	1st Instar	2nd Instar	3rd Instar	4th Instar	5th Instar	Female	Male
1	1	5	5	5	5	5	5
2	3	10	10	10	10	10	10
3	5	15	15	15	15	20	20
4	8	20	20	20	20	30	30
5	10	30	30	30	30	60	60

**Table 2:** Functional response parameters of the *Tytthus chinensis* to *Sogatella furcifera* eggs.

Developmental Stage	*r* ^2^	Attack Rate ± SE (*a*)	Handling Time ± SE (*T_h_*)	Theoretical Daily Maximum Prey Consumption	Predation Capacity (*a*/*T_h_*)
1st instar	0.938	0.987 ± 0.0.264	0.138 ± 0.041	7.25	7.15
2nd instar	0.972	0.653 ± 0.067	0.109 ± 0.008	9.17	5.99
3rd instar	0.921	0.882 ± 0.203	0.043 ± 0.014	23.26	20.51
4th instar	0.970	1.659 ± 0.348	0.032 ± 0.008	31.25	51.84
5th instar	0.997	3.108 ± 0.309	0.029 ± 0.002	34.48	107.17
Female	0.989	2.242 ± 0.299	0.024 ± 0.002	41.67	93.42
Male	0.985	11.354 ± 1.211	0.053 ± 0.006	18.87	214.23

Note: The theoretical daily maximum prey consumption was calculated as 1/*T_h_*.

**Table 3:** Development and fecundity of *Tytthus chinensis* under different densities of *Sogatella furcifera* eggs.

Parameters		Prey Density (Eggs/Day)			
		4	10	20	30
Nymphal duration (days)	1st instar	2.59 ± 0.13 b	2.63 ± 0.16 ab	3.00 ± 0.15 a	2.60 ± 0.12 b
	2nd instar	2.00 ± 0.68 a	2.07 ± 0.11 a	2.07 ± 0.09 a	2.27 ± 0.64 a
	3rd instar	2.29 ± 0.62 a	2.28 ± 0.14 a	2.04 ± 0.15 a	1.93 ± 0.13 b
	4th instar	2.33 ± 0.14 a	2.17 ± 0.15 a	2.00 ± 0.12 a	2.10 ± 0.10 a
	5th instar	2.38 ± 0.15 a	2.45 ± 0.19 a	2.59 ± 0.20 a	2.59 ± 0.12 a
Pre-adult duration (days)		11.50 ± 0.22 a	11.72 ± 0.30 a	11.55 ± 0.25 a	11.37 ± 0.25 a
Pre-oviposition period (APOP) (days)		4.00 ± 0.28 a	3.36 ± 0.02 a	3.17 ± 0.07 a	3.67 ± 0.01a
Male longevity (days)		18.33 ± 3.49 a	20.73 ± 3.14 a	19.40 ± 3.29 a	14.56 ± 3.25 a
Female longevity (days)		12.75 ± 3.45 a	14.64 ± 0.90 a	14.83 ± 0.86 a	14.33 ± 1.51 a
Fecundity (eggs/female)		8.00 ± 2.40 b	30.10 ± 3.70 a	31.42 ± 3.08 a	31.06 ± 2.99 a
Egg duration of the next generation		8.10 ± 0.16 a	8.10 ± 0.14 a	8.06 ± 0.15 a	8.00 ± 0.15 a
Hatchingn rate of the next generation		53.13 ± 5.98% b	87.50 ± 5.10% a	96.88 ± 3.13% a	96.88 ± 3.13% a

Note: The data in the table are presented as the mean ± standard error. At a prey density of 4 eggs/day, female adults laid a total of 32 eggs, which were subsequently used to calculate the egg period and hatching rate. Standard errors were estimated using 100,000 bootstrap replicates, except for egg duration and hatching rate of the next generation, the means and errors of which were determined using the LSD method. Means labeled with different letters within each row indicate statistically significant differences among the four prey density treatments, as determined by the paired bootstrap test at the 5% significance level.

**Table 4:** Life table parameters of *Tytthus chinensis* under different *Sogatella furcifera* egg densities.

Prey Density (Eggs/Day)	Net Reproductive Rate (*R*_0_)	Mean GENERATION Time (*T*)	Intrinsic Rate of Increase (*r_m_*)	Finite Rate of Increase (*λ*)	Sex Ratio (%)
4	1.07 ± 0.56 b	25.15 ± 2.56 a	0.003 ± 0.02 c	1.00 ± 0.032 c	25%
10	11.03 ± 2.96 a	25.83 ± 0.43 a	0.09 ± 0.01 b	1.10 ± 0.01 b	50%
20	12.57 ± 3.05 a	25.07 ± 0.51 ab	0.10 ± 0.01 ab	1.11 ± 0.01 ab	66.67%
30	18.63 ± 3.29 a	24.25 ± 0.40 b	0.12 ± 0.01 a	1.13 ± 0.01 a	66.67%

Note: Standard errors were estimated based on 100,000 bootstrap replicates. Means labeled with different letters within each column indicate statistically significant differences among the four prey density treatments, as determined by the paired bootstrap test at the 5% significance level.

## Data Availability

The original contributions presented in this study are included in the article. Further inquiries can be directed to the corresponding author.
